# A phase 1, randomized, double-blind, placebo-controlled, dose escalation study to evaluate the safety, tolerability, pharmacokinetics and immunogenicity of SHR-1905, a long-acting anti-thymic stromal lymphopoietin antibody, in healthy subjects

**DOI:** 10.3389/fphar.2024.1400696

**Published:** 2024-07-15

**Authors:** Yue Fei, Na Li, Weilin Qian, Yang Fan, Yu Shen, Quanren Wang, Kristi McLendon, Kai Shen

**Affiliations:** ^1^ Jiangsu Hengrui Pharmaceuticals Co., Ltd., Shanghai, China; ^2^ Nucleus Network Pty Ltd, Brisbane, QLD, Australia

**Keywords:** asthma, thymic stromal lymphopoietin, phase 1, pharmacokinetics, safety

## Abstract

**Introduction:**

Thymic stromal lymphopoietin (TSLP) is integral to inducing innate and T helper two cell inflammation that leads to clinical symptoms of asthma. SHR-1905 is a humanized immunoglobulin G1 kappa monoclonal antibody that inhibits TSLP bioactivity, developed for the treatment of severe uncontrolled asthma. This phase 1, randomized, double-blind, placebo-controlled single ascending dose study assessed the safety, tolerability, pharmacokinetics (PK), and immunogenicity of subcutaneous SHR-1905 in healthy subjects.

**Methods:**

Five dose cohorts were planned (50, 100, 200, 400, and 600 mg) and subjects were randomized (8:2) in each cohort to receive SHR-1905 or placebo with a follow-up period up to Day 253.

**Results:**

The majority of treatment-emergent adverse events (TEAEs) were mild and the incidence of TEAEs was comparable between the SHR-1905 and the placebo groups. The maximum serum concentration was reached 7.0–17.6 days after injection. The serum concentration of SHR-1905 increased with increasing dose level, and SHR-1905 exposure exhibited in a slightly greater-than-dose-proportional manner from 50 to 600 mg. SHR-1905 had a prolonged serum half-life around 80 days supporting every 6-month dosing. In SHR-1905 treated subjects, 15% tested positive for anti-drug antibodies post-dose with no apparent effect on corresponding PK profiles or safety.

**Conclusion:**

SHR-1905 demonstrated a good safety and tolerability profile with a long half-life in healthy subjects after a single administration in the dose range of 50–600 mg.

**Clinical Trial Registration:**

clinicaltrials.gov, identifier NCT04800263

## 1 Introduction

Asthma is a common chronic respiratory disease characterized by symptoms of wheezing, tachypnea, chest tightness, and cough (Papi et al., 2018). There are approximately 339 million asthma patients worldwide, of which 5%–10% have severe asthma with poorly controlled conditions despite high-dose standard of care (Lang, 2015; Cottini et al., 2021). The persistence of symptoms and exacerbations (which can be life-threatening) lead to overall deterioration and dramatically affects quality of life.

Attenuation of inflammation has always been an important direction for asthma treatment. At least half of patients with severe asthma are type 2 (T2) inflammation-driven (Habib et al., 2022; Thio and Chang, 2023). Thymic stromal lymphopoietin (TSLP) is an epithelial interleukin (IL)-7-like cytokine, acting as an alarmin upstream of the T2 inflammatory pathway (Matera et al., 2020). TSLP receptor complex consists of TSLP receptor (TSLPR) and IL-7Rα (Nian et al., 2019). TSLP first binds to TSLPR with a relatively low affinity and recruits IL-7Rα with high affinity (Nian et al., 2019; Matera et al., 2020). The binding induces the activation of signal pathways such as signal transducer and activator of transcription 5, leading to the maturation of dendritic cells (DCs) and the differentiation of T cells (Nian et al., 2019; Matera et al., 2020). Specifically, TSLP-activated myeloid DCs secrete cytokines including IL-8, eotaxin-2, thymus and activation regulated chemokine, and macrophage-derived chemokine, and highly express OX40L (Liu, 2006; Liu, 2009). In the absence of T helper one-polarizing cytokine IL-12, OX40L binds to naïve CD4^+^ T cells, making them differentiate into T helper two cells (Liu, 2006; Liu, 2009). T helper two cells then secrete T2 cytokines and induce T2 inflammatory response in the body (Liu, 2006; Liu, 2009). Eventually, this leads to pathological conditions of severe asthma and even glucocorticoid tolerance.

TSLP also acts on group 2 innate lymphoid cells (ILC2s), which plays an important role in both T2 and innate inflammation (Menzies-Gow et al., 2020). When exposed to epithelial insults like viruses, bacteria, and pollutants, TSLP, in combination with IL-33, activates ILC2s to produce T2 cytokines including IL-5 and IL-13 (10). In this way, ILC2s can drive T2 immune responses in absence of T cells (Matsuyama et al., 2022). ILC2 production of T2 cytokines also promotes eosinophilic inflammation (Gauvreau et al., 2020; Menzies-Gow et al., 2020; Matsuyama et al., 2022). Meanwhile, TSLP improves the survival and longevity of ILC2s, thus prolonging the effect of ILC2 in asthma pathogenesis (Porsbjerg et al., 2020). In terms of innate immune responses, TSLP can induce DCs to directly recruit neutrophils and eosinophils, resulting in neutrophilic and eosinophilic inflammation (Gauvreau et al., 2020; Ebina-Shibuya and Leonard, 2023). TSLP also acts on mast cells and natural killer cells, and mediates innate inflammation by producing IL-4, IL-6, and immunoglobulin (Ig) E (Gauvreau et al., 2020; Ebina-Shibuya and Leonard, 2023). In summary, TSLP can induce innate and T2 inflammation simultaneously, thereby increasing tissue mucus, airway remodeling, and severe cell fibrosis, and gradually evolving into asthma (Liu, 2006; Liu, 2009; Gauvreau et al., 2020; Ebina-Shibuya and Leonard, 2023).

Currently, there are several anti-TSLP monoclonal antibodies under development (Matera et al., 2020; O’Byrne et al., 2023), in which tezepelumab demonstrated positive results in treating patients with poorly controlled severe asthma in the phase 3 clinical trial (Menzies-Gow et al., 2021). SHR-1905 is a humanized anti-TSLP monoclonal antibody (IgG1 kappa subtype) with an optimized molecular design. It can bind to TSLP and block the interaction between TSLP and its receptor complex, thereby preventing TSLP-targeted immune cells from releasing proinflammatory cytokines to prevent asthma attack and improve asthma control. Since TSLP acts on the early upstream of the inflammatory cascade, SHR-1905 may be suitable for a wide range of patients with severe uncontrolled asthma, potentially including those with non-T2-driven asthma. This first-in-human phase 1 study aimed to evaluate the safety, tolerability, pharmacokinetics (PK), and immunogenicity of SHR-1905 in healthy subjects.

## 2 Materials and methods

### 2.1 Study design

This was a single-center, randomized, double-blind, placebo-controlled, single ascending dose, phase 1 study in healthy subjects conducted in Australia from 16 July 2021 to 12 November 2022 (ClinicalTrials.gov: NCT04800263). Overall, five dose cohorts were planned, including 50, 100, 200, 400, and 600 mg. For each cohort, ten subjects were enrolled with eight randomized to receive SHR-1905 and two randomized to receive placebo.

Dose escalation was decided by the Safety Monitoring Committee based on the review of the available safety data at least 15 days after administration in the preceding lower dose cohort. Each cohort began with two sentinel subjects, one in SHR-1905 group and one in placebo group. Once the dose was deemed to be safe and well-tolerated by the investigator and the sponsor after 48 h post-dose, the remaining eight subjects were dosed. Subjects were administered a single dose of subcutaneous SHR-1905 or placebo on Day 1 and closely monitored in the hospital for 5 days post-dosing. On Day 6, subjects were discharged and were required a safety follow-up visit starting from Day 8. Based on the observed longer half-life of SHR-1905, the safety follow-up was approved to be extended from up to Day 113 to Day 253.

The protocol and all amendments were approved by the Ethics Committee at the study center. The study was conducted according to the Declaration of Helsinki, Guidelines for Good Clinical Practice, and local laws and regulations. All subjects provided written informed consent.

### 2.2 Study population

Eligible subjects were males and females aged between 18 and 55 years (inclusive) with a total body weight ≥45.0 kg, a body mass index (BMI) between 18.0 and 30.0 kg/m^2^ (inclusive), and no clinically significant abnormalities in medical history, general physical examination, vital signs, laboratory tests, and 12-lead electrocardiogram (ECG). Subjects were excluded if they had a known history or were suspected of being allergic to anti-TSLP antibodies, their formulation excipients, or other biologic products and drugs; participated in clinical trials of other investigational drugs or medical devices within 3 months prior to screening or within five half-lives of any drugs during screening visit, or in the follow-up period of a clinical study; or had live (attenuated) vaccination within 1 month before screening or plan to be vaccinated of live (attenuated) vaccine during the trial.

### 2.3 Randomization and blinding

The randomization code was generated by statistical software and loaded into the Randomization and Trial Supply Management system. The SHR-1905 and placebo injections were identical in size, color, and transparency. Subjects, investigators, and sponsor study team were blinded for the treatment allocation.

### 2.4 Safety and tolerability assessment

Safety assessments included incidence and severity of adverse events (AEs) and serious AEs (SAEs), vital signs, physical examination, laboratory tests, 12-lead ECG, and injection site examination. Subjects were monitored for AEs and SAEs at all study visits. AEs were classified according to System Organ Class and Preferred Term using the Medical Dictionary for Regulatory Activities (version 25.0) and AE severity was graded mild, moderate, and severe.

### 2.5 Pharmacokinetics assessment

Serum samples for PK measurements were collected at pre-dose (within 60 min before dosing), at 12, 24, 48, 72, 96, and 120 h after dosing, and on Days 8, 11, 15, 22, 29, 43, 57, 71, 85, 113, 141, 197, and 253 or withdrawal. Samples were analyzed using a validated enzyme-linked immunosorbent assay (ELISA) to determine the concentration of SHR-1905 at Accurant BioTech (Shanghai, China). Briefly, anti-SHR-1905 antibodies were coated on 96-well ELISA plate, and the SHR-1905 bound to the anti-SHR-1905 antibodies following incubation and then detected by horseradish peroxidase conjugated anti-Human IgG1. The lower limit of quantitation of the assay was 100 ng/mL and the upper limit of quantitation was 2,400 ng/mL.

### 2.6 Immunogenicity assessment

Serum samples were collected at pre-dose (within 60 min before dosing) and on Days 8, 15, 29, 57, 85, 113, 197, and 253 or withdrawal for anti-drug antibody (ADA) detection. Samples were analyzed to detect anti-SHR-1905 antibodies using a validated electrochemiluminescence assay at Accurant BioTech (Shanghai, China). The ADA assay was based on an affinity capture bridging assay design. The positive control samples were prepared by spiking anti-SHR-1905 antibodies in human serum. The assay sensitivity, low positive control, and titer range were established as 7.88 ng/mL, 50.00 ng/mL, and 69–624, respectively. No assay interference was observed at TSLP concentrations of 30.00 ng/mL. No hook effect was observed.

ADA-positive responses were designated as treatment-induced or treatment-boosted. Treatment-induced ADA-positive subject was defined as a subject who had a baseline ADA-negative sample and at least one post-baseline ADA-positive sample, while treatment-boosted ADA-positive subject was defined as a subject who had both baseline and post-baseline ADA-positive samples, and the titer of the post-baseline sample was equal to or more than 4-fold of the baseline titer.

### 2.7 Statistical analysis

Safety was assessed in all subjects who were randomized and received one dose of SHR-1905 or placebo. PK parameters were assessed in all randomized and SHR-1905 dosed subjects who had at least one assessment for PK concentration or parameter. Immunogenicity was assessed in all randomized subjects who received one dose of SHR-1905 or placebo, and had baseline and at least one post-baseline assessment for ADA evaluation.

Safety data, PK parameters, and immunogenicity results were summarized by treatment groups using descriptive statistics. The serum concentration-time curves of SHR-1905 were plotted by dose. PK parameters were calculated, including maximum observed concentration (C_max_), time of maximum observed concentration (T_max_), area under the curve from the time of dosing to the last measurable concentration (AUC_last_), area under the curve from the time of dosing extrapolated to infinity (AUC_0-inf_), terminal elimination half-life (t_1/2_), apparent clearance (CL/F), and apparent volume of distribution (V/F). Dose-exposure proportionality over the range of tested dose levels was assessed using PK parameters AUC_last_, AUC_0-inf_, and C_max_. A linear fitting between log(PK parameters) and log(dose) was conducted using power model, and the estimates of slope and the 2-sided 90% confidence intervals (CIs) were provided. PK parameters were calculated using non-compartmental methods by Phoenix WinNonlin (Version 8.3, Certara USA, Inc.). Other statistical analyses were conducted using SAS (Version 9.4, SAS Institute, Inc.).

## 3 Results

### 3.1 Study population

A total of 50 healthy subjects were randomized and assigned to SHR-1905 or placebo in five ascending dose cohorts (40 received SHR-1905 and 10 received placebo). All 50 enrolled subjects completed the administration at the dose planned, while 49 subjects completed the entire safety follow-up as scheduled (9 up to Day 113 and 40 up to Day 253; Supplementary Table S1; Supplementary Figure S1). One subject in the 200 mg cohort, whose follow-up was planned up to Day 253, withdrew from the study after completing the Day 113 follow-up due to personal reasons.

Of all enrolled subjects, 22 (44.0%) were male and 28 (56.0%) were female. The majority of subjects were Caucasians (80.0%). The mean (standard deviation) age was 29.1 (9.1) years, and the mean (standard deviation) BMI was 23.4 (3.0) kg/m^2^. Among all 50 subjects, 35 (70%) had medical histories, but none of which were clinically significant at baseline. There were 20 (40%) subjects who had at least one prior medication and 42 (84%) subjects who were on at least one concomitant medication. The concomitant medications used in this study were mainly for contraception, COVID-19 prophylaxis, medical history treatment, or AE treatment. None of the prior or concomitant medications were considered to have an impact on the study drug or the study results. The demographics and baseline characteristics were comparable across different doses of SHR-1905 and placebo groups (Table 1).

**TABLE 1:** Baseline demographics and characteristics.

	Placebo (n = 10)	SHR-1905					
		50 mg (n = 8)	100 mg (n = 8)	200 mg (n = 8)	400 mg (n = 8)	600 mg (n = 8)	Total (n = 40)
Age (year), mean (SD)	28.4 (8.7)	29.0 (5.0)	28.6 (10.7)	32.4 (12.4)	28.1 (11.3)	28.4 (7.3)	29.3 (9.4)
Sex, n (%)							
Male	4 (40.0)	7 (87.5)	4 (50.0)	2 (25.0)	3 (37.5)	2 (25.0)	18 (45.0)
Female	6 (60.0)	1 (12.5)	4 (50.0)	6 (75.0)	5 (62.5)	6 (75.0)	22 (55.0)
Race, n (%)							
American Indian or Alaska Native	1 (10.0)	1 (12.5)	0	0	1 (12.5)	0	2 (5.0)
Asian	1 (10.0)	2 (25.0)	1 (12.5)	0	1 (12.5)	2 (25.0)	6 (15.0)
White	8 (80.0)	5 (62.5)	7 (87.5)	8 (100.0)	6 (75.0)	6 (75.0)	32 (80.0)
BMI (kg/m^2^), mean (SD)	22.9 (3.7)	25.7 (3.1)	23.0 (2.1)	22.3 (1.6)	23.2 (3.7)	23.5 (2.6)	23.5 (2.8)
Medical history, n (%)	7 (70.0)	8 (100.0)	4 (50.0)	6 (75.0)	6 (75.0)	4 (50.0)	28 (70.0)
Prior medications, n (%)	4 (40.0)	2 (25.0)	3 (37.5)	4 (50.0)	2 (25.0)	5 (62.5)	16 (40.0)
Concomitant medications, n (%)	9 (90.0)	5 (62.5)	8 (100.0)	7 (87.5)	6 (75.0)	7 (87.5)	33 (82.5)

BMI, Weight (kg)/Height^2^ (m^2^); BMI, body mass index; SD, standard deviation.

### 3.2 Safety and tolerability

All of the 50 treated subjects were included in the safety analysis set. A summary of AEs in the study is presented in Table 2. Treatment-emergent adverse events (TEAEs) were reported in 39 subjects (78.0%), including 33 (82.5%) in the SHR-1905 treatment groups and 6 (60.0%) in the placebo group. A total of 20 subjects (40.0%) had at least one treatment-related adverse event (TRAE) during this study, including 18 (45.0%) in the SHR-1905 treatment groups and 2 (20.0%) in the placebo group. The majority of TEAEs were mild in severity while five subjects (12.5%) in the SHR-1905 groups and one subject (10.0%) in the placebo group reported TEAEs that were moderate in severity, among which, a papular rash was reported in the 100 mg SHR-1905 group that was regarded as possibly-related to the study drug. There were no SAEs that occurred and no TEAEs leading to study discontinuation or death during the study.

**TABLE 2:** Summary of adverse events.

	Placebo (n = 10)	SHR-1905						Overall (n = 50)
		50 mg (n = 8)	100 mg (n = 8)	200 mg (n = 8)	400 mg (n = 8)	600 mg (n = 8)	Total (n = 40)	
Subjects with at least one TEAE	6 (60.0)	5 (62.5)	7 (87.5)	7 (87.5)	7 (87.5)	7 (87.5)	33 (82.5)	39 (78.0)
Mild	6 (60.0)	5 (62.5)	6 (75.0)	7 (87.5)	7 (87.5)	7 (87.5)	32 (80.0)	38 (76.0)
Moderate	1 (10.0)	0	1 (12.5)	1 (12.5)	1 (12.5)	2 (25.0)	5 (12.5)	6 (12.0)
Severe	0	0	0	0	0	0	0	0
Subjects with at least one TRAE	2 (20.0)	2 (25.0)	4 (50.0)	4 (50.0)	3 (37.5)	5 (62.5)	18 (45.0)	20 (40.0)
Mild	2 (20.0)	2 (25.0)	3 (37.5)	4 (50.0)	3 (37.5)	5 (62.5)	17 (42.5)	19 (38.0)
Moderate	0	0	1 (12.5)	0	0	0	1 (2.5)	1 (2.0)
Severe	0	0	0	0	0	0	0	0
Most common TEAEs^a^								
Headache	4 (40.0)	2 (25.0)	1 (12.5)	2 (25.0)	2 (25.0)	3 (37.5)	10 (25.5)	14 (28.0)
COVID-19	1 (10.0)	0	2 (25.0)	2 (25.0)	3 (37.5)	3 (37.5)	10 (25.0)	11 (22.0)
Back pain	0	0	0	2 (25.0)	1 (12.5)	2 (25.0)	5 (12.5)	5 (10.0)
Dermatitis contact	0	2 (25.0)	0	1 (12.5)	0	1 (12.5)	4 (10.0)	4 (8.0)
Upper respiratory tract infection	1 (10.0)	0	0	0	1 (12.5)	2 (25.0)	3 (7.5)	4 (8.0)
Troponin I increased	0	1 (12.5)	1 (12.5)	0	1 (12.5)	0	3 (7.5)	3 (6.0)
Immunization reaction	0	1 (12.5)	0	0	1 (12.5)	1 (12.5)	3 (7.5)	3 (6.0)
Injection site erythema	1 (10.0)	0	0	1 (12.5)	0	0	1 (2.5)	2 (4.0)
Palpitations	1 (10.0)	0	0	1 (12.5)	0	0	1 (2.5)	2 (4.0)
Presynocope	1 (10.0)	0	0	1 (12.5)	0	0	1 (2.5)	2 (4.0)
Concussion	1 (10.0)	0	0	0	0	0	0	1 (2.0)
Fatigue	1 (10.0)	0	0	0	0	0	0	1 (2.0)
Influenza like illness	1 (10.0)	0	0	0	0	0	0	1 (2.0)
Nasal congestion	1 (10.0)	0	0	0	0	0	0	1 (2.0)
Nausea	1 (10.0)	0	0	0	0	0	0	1 (2.0)

TEAE, treatment-emergent adverse event; TRAE, treatment-related adverse event. Data presented are n (%).

^a^ Most common TEAEs, were TEAEs, that occurred in >5% of subjects in SHR-1905; treatment groups or placebo group.

The most common (>5%) TEAEs in the SHR-1905 treatment groups were headache (25.5%), COVID-19 (25.0%), backpain (12.5%), contact dermatitis (10.0%), upper respiratory tract infection (7.5%), increased troponin I (7.5%), and immunization reaction (7.5%), while the most common TEAEs in the placebo group were headache (40.0%), and COVID-19, upper respiratory tract infection, injection site erythema, palpitations, presyncope, concussion, fatigue, influenza like illness, nasal congestion and nausea with an incidence of 10% (Table 2). There was no apparent trend of dose-dependent TEAE or dose-dependent TRAE.

No clinically significant abnormality of vital signs and 12-lead ECG was observed in study subjects. The laboratory parameters in most subjects were normal at baseline and post-dose. All elevated troponin I events resolved within 1–2 days without medication or therapy, while no apparent association was identified with ECG abnormalities. The increases of creatine kinase observed were deemed not treatment-related and resolved without medication or therapy. Injection site reactions were observed in six subjects (15.0%) in the SHR-1905 treatment group and one subject (10.0%) in the placebo group. All the injection site reactions were classified as mild in severity and possibly/certainly related to study treatment, all of which recovered without intervening treatment. There was no apparent dose-related trend among SHR-1905 treatment groups in the mean values of laboratory parameters and the incidence of injection site reaction.

### 3.3 Pharmacokinetics

**FIGURE 1:**
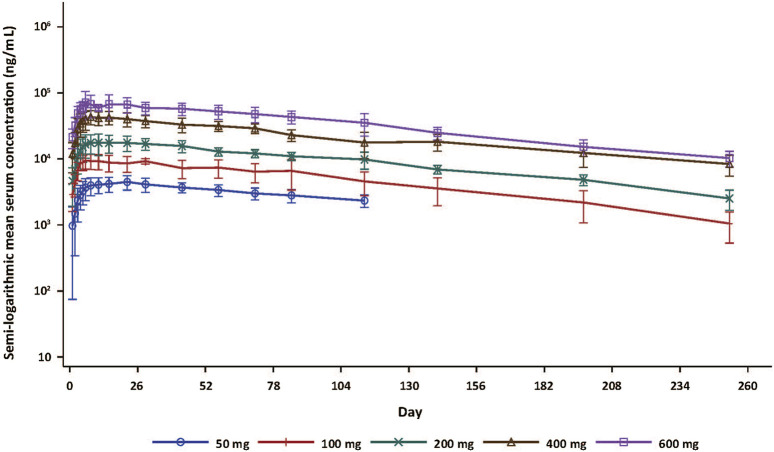
Serum concentration-time curve (semi-log) following SHR-1905 administration in healthy subjects. Error bars show standard deviation.

Forty subjects who received SHR-1905 were included in the PK analysis. The serum SHR-1905 concentration-time profiles of SHR-1905 by dose are presented in Figure 1. The serum concentrations of SHR-1905 increased along with the increased dose level from 50 to 600 mg. The PK parameters following SHR-1905 administration are summarized in Table 3. The median T_max_ was between 7.0 and 17.6 days after the injection. The sensitivity analysis of T_max_, after excluding the two outliers at 100 mg and 400 mg, showed the same median T_max_ with decreased variability. The mean t_1/2_ was around 80 days across the dose range. The geomean CL/F was comparable across different doses, ranging from 0.0621 to 0.09 L/day. The geomean V/F ranged from 7.41 to 9.12 L. Both C_max_ and AUC_0-inf_ increased with increased doses from 50 to 600 mg. The slopes of C_max_, AUC_last_, and AUC_0-inf_ in power model analysis were 1.129 (90% CI, 1.043, 1.216), 1.182 (90% CI, 1.086, 1.279), and 1.092 (90% CI, 0.956, 1.227), respectively (Supplementary Table S2), indicating that the exposure of SHR-1905 appears to increase in a slightly greater-than-dose-proportional manner with increased dose.

**TABLE 3:** Summary of SHR-1905 pharmacokinetics parameters.

	SHR-1905				
	50 mg (N = 8)	100 mg (N = 8)	200 mg (N = 8)	400 mg (N = 8)	600 mg (N = 8)
C_max_, μg/mL	4.4 (30)	9.4 (40)	18.7 (30)	43.6 (20)	74.2 (40)
T_max_, day	17.6 (5.0, 28.2)	7.0 (4.0, 84.0)^e^	11.9 (5.0, 28.0)	7.1 (5.0, 70.0)^e^	7.1 (5.0, 26.1)
t_1/2_, day	84.9 (7)^a^	69.7 (20)^b^	78.6 (10)^c^	77.3 (20)^d^	83.3 (10)
AUC_last_, day·μg/mL	445 (40)	1000 (60)	2110 (20)	5130 (20)	8450 (20)
AUC_0-inf_, day·μg/mL	801 (20)^a^	1110 (60)^b^	2480 (20)^c^	5640 (20)^d^	9670 (20)
CL/F, L/day	0.0624 (20)^a^	0.0900 (60)^b^	0.0807 (20)^c^	0.0709 (20)^d^	0.0621 (20)
V/F, L	7.64 (10)^a^	8.91 (50)^b^	9.12 (10)^c^	7.81 (7)^d^	7.41 (20)

T_max_ was presented in Median (range); t_1/2_ was presented in Mean (%CV); other parameters were presented in Geomean (%GeoCV). AUC_last_, area under the curve from the time of dosing to the last measurable concentration; AUC_0-inf_, area under the curve from the time of dosing extrapolated to infinity; CL/F, apparent clearance; C_max_, maximum observed concentration; T_max_, time of maximum observed concentration; t_1/2_, terminal elimination half-life; V/F, and apparent volume of distribution. %CV, coefficient of variation; %GeoCV, geometric coefficient of variation.

a-d PK, parameters t_1/2_, AUC_0-inf_, CL/F, and V/F were affected by the terminal elimination rate constant (λ_z_). Subjects whose PK results did not meet the quality control standards for the estimation of λ_z_ were excluded from the analyses for t_1/2_, AUC_0-inf_, CL/F, and V/F.

^a^ n = 3; only three subject values in the 50 mg cohort were available for statistical analysis.

^b^ n = 7; only seven subject values in the 100 mg cohort were available for statistical analysis.

^c^ n = 5; only five subject values in the 200 mg cohort were available for statistical analysis.

^d^ n = 4; only four subject values in the 400 mg cohort were available for statistical analysis.

^e^ T_max_ was 7.0 (4.0, 13.9) days in 100 mg and 7.1 (5.0, 21.0) days in 400 mg after excluding the outliers in these two cohorts, respectively (n = 7 in each cohort).

### 3.4 Immunogenicity

**FIGURE 2:**
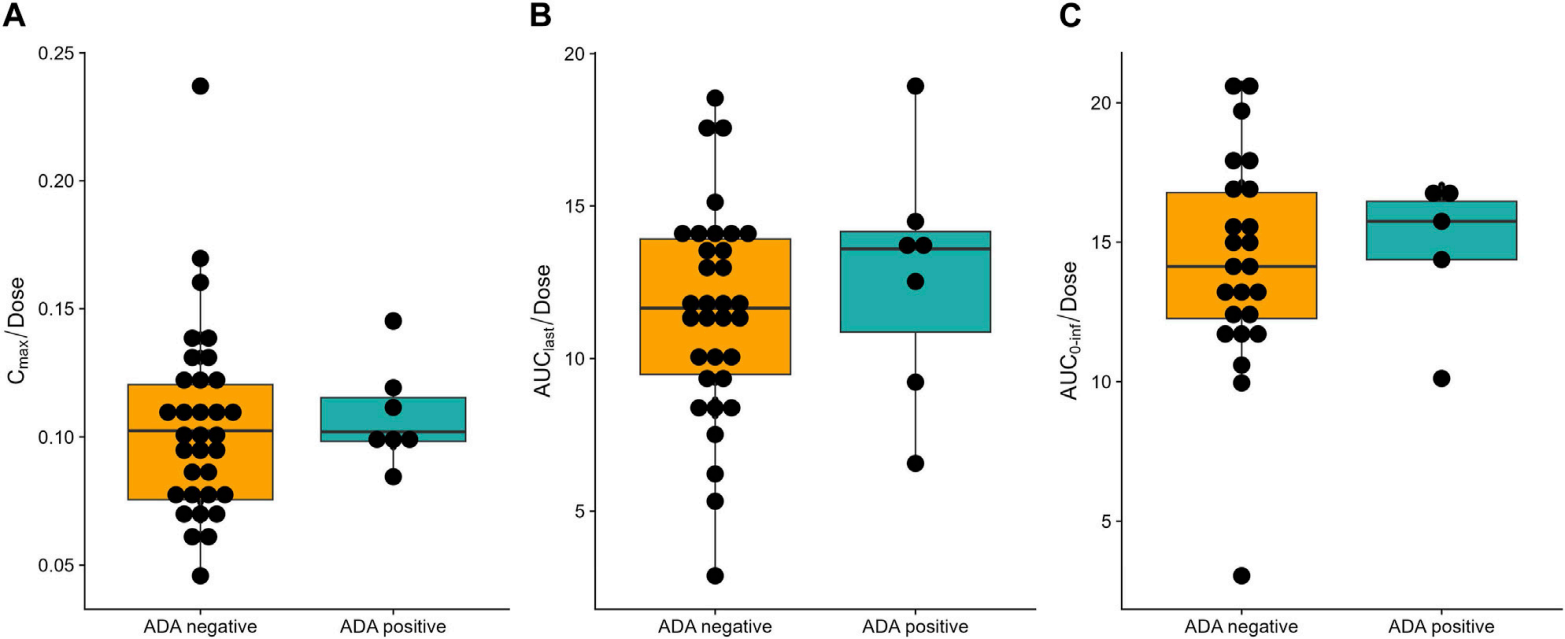
Influence of anti-drug antibody (ADA) on dose normalized **(A)** C_max_, **(B)** AUC_last_, **(C)** AUC_0-inf_ of SHR-1905. Boxplot of PK profiles of ADA-positive and ADA-negative subjects.

All 50 subjects were included in the ADA analysis (Supplementary Table S3). Treatment-induced ADA positivity was reported in both placebo group (1 [10.0%]) and SHR-1905 treatment groups (6 [15.0%] overall; 1 [12.5%] in 200 mg cohort, 2 [25.0%] in 400 mg cohort, and 3 [37.5%] in 600 mg cohort). The observed earliest time point of treatment-induced ADA after SHR-1905 treatment was Day 113 for 200 and 600 mg cohorts and Day 197 for 400 mg cohort. No treatment-boosted ADA-positive response was observed in this study. In addition, there were no obvious differences in the corresponding PK profiles between the ADA-positive and ADA-negative subjects (Figure 2). No apparent influence of ADA positive was found on subjects’ safety profiles.

## 4 Discussion

In this first-in-human study, SHR-1905, a long-acting anti-TSLP antibody, demonstrated a good safety and tolerability profile in healthy subjects in the dose range of 50–600 mg. No SAEs were reported and no TEAEs resulted in study discontinuation or death during the study. The majority of TEAEs were mild. TEAEs and TRAEs were evenly distributed in each dose cohort, and the incidence of TEAEs was relatively comparable between the SHR-1905 and the placebo groups. There were no apparent dose-dependent trends among different SHR-1905 dose cohorts in TEAEs and TRAEs incidences. The study was conducted during the COVID-19 pandemic and, as noted, 25% of SHR-1905 treated subjects in this study were positive for this infection, which might be a possible explanation for the transient troponin I and creatine kinase elevations for those subject with COVID-19 positive prior to these AEs. One moderate AE, namely, papular rash, was reported to be possibly-related to SHR-1905 treatment. A more definitive relationship between this AE and SHR-1905 needs to be further explored in subsequent studies.

Overall, the PK characteristics of SHR-1905 were favorable. Following administration of a single injection of SHR-1905 from 50 to 600 mg, the concentration as well as total exposure of SHR-1905 increased along with increased dose levels. The power model analysis of SHR-1905 demonstrated a slightly greater-than-dose-proportional manner over the dose range investigated. PK parameters V/F and CL/F of SHR-1905 demonstrated similar characteristics as those in the phase 1 trial of tezepelumab and other therapeutic monoclonal antibodies (Dirks and Meibohm, 2010; Keizer et al., 2010; Sakamoto et al., 2020). The relatively frequent COVID-19 events did not show any significant impact on the PK profiles of SHR-1905. Notably, the half-life of SHR-1905 was about 3 times longer than that of tezepelumab (mean, 69.7–84.9 days for SHR-1905 vs. mean, 23.9–26.3 days in tezepelumab) (Sakamoto et al., 2020). This is attributed to the molecular modification of SHR-1905, in which the Fc segment of the antibody has been modified through amino acid mutation. The Fc segment plays an important role in the elimination of monoclonal antibodies, where the binding of IgG to the neonatal Fc receptor protects monoclonal antibodies from lysosomal degradation (Hinton et al., 2004; Tabrizi et al., 2006; Sakamoto et al., 2020). The mutation of Fc segment increases the binding affinity of the antibody for Fc receptor, thus reducing the elimination of antibodies in humans and extending the antibody half-life (Hinton et al., 2004; Tabrizi et al., 2006; Sakamoto et al., 2020). The longer half-life of SHR-1905 allows a lower frequency of administration in clinical practice. Given such a long half-life, there was a longer absorption phase and a relatively steady decrease of SHR-1905 concentrations after T_max_. The higher SHR-1905 T_max_ variability within subjects and across administration groups in this study was mainly driven by the two outliers in 100 mg and 400 mg cohorts, respectively. After excluding the outliers, T_max_ variability of SHR-1905 was largely decreased and relatively comparable between the five dose cohorts, which was also similar to that of Tezepelumab (2.99–21.25 days and 1.00–14.02 days of accessorized prefilled syringe and autoinjector, respectively) in healthy participants (Zheng et al., 2021).

Treatment-induced ADAs were found in 10.0% (1/10) of placebo-treated subjects and 15.0% (6/40) of SHR-1905-treated subjects. The treatment-induced ADA response in the subject receiving placebo may be due to environmental exposure or auto-antibodies (Gorovits et al., 2016). The immunogenicity of SHR-1905 is comparable to that of other monoclonal antibodies for the treatment of asthma such as mepolizumab, reslizumab, benralizumab, and depemokimab. The incidence of ADA ranged from 5% to 9% for mepolizumab (Ortega et al., 2014; Lugogo et al., 2016). About 5.4% of participants tested positive for ADA in the phase 3 reslizumab study (Zou et al., 2018). Benralizumab and depemokimab have higher ADA-positive rates, which are 13.9% and 25%, respectively (Singh et al., 2022; Cheung et al., 2023). Importantly, although SHR-1905 demonstrated certain immunogenicity, no obvious effect of ADA positivity on SHR-1905 exposure or AE incidence was observed, and the earliest time to positive ADA was 113 days. The longevity of the ADA response was not estimated due to the limited follow-up period. In addition, neutralizing properties of the ADA response should be explored in future studies.

One limitation of the study is the relatively small sample size, in which less common adverse effects would be more difficult to observe. No pharmacodynamic markers of disease were investigated. Future studies will be carried out in the target population to further assess the efficacy of SHR-1905. Additionally, all the subjects were followed for only three half-lives (253 days) of SHR-1905 in this study due to a quite long follow-up period of five half-lives, which might give a shorter t_1/2_ observed.

## 5 Conclusion

A single subcutaneous dose of SHR-1905 up to 600 mg demonstrated a good safety and tolerability profile in healthy subjects, along with a longer half-life supporting every 6-month dosing. Our findings support the multi-center, randomized, double-blind, placebo-controlled phase 2 study of SHR-1905 in severe uncontrolled asthma (ClinicalTrials.gov, NCT05593250).

## Data Availability

The raw data supporting the conclusions of this article will be made available by the authors, without undue reservation.
