# Supplementary material for: A phase 1, randomized, double-blind, placebo-controlled, dose escalation study to evaluate the safety, tolerability, pharmacokinetics and immunogenicity of SHR-1905, a long-acting anti-thymic stromal lymphopoietin antibody, in healthy subjects
Source: Front Pharmacol. 2024 Jul 15;15:1400696. doi: 10.3389/fphar.2024.1400696 (PMC11284144; doi:10.3389/fphar.2024.1400696)
Supplement: Supplementary file 1 [file Table1.docx]

**Table S1. Subject Disposition**

|  | Placebo | SHR-1905 | | | | | | Overall |
| --- | --- | --- | --- | --- | --- | --- | --- | --- |
|  |  | 50 mg | 100 mg | 200 mg | 400 mg | 600 mg | Total |  |
| No. of subjects screened |  |  |  |  |  |  |  | 179 |
| No. of subjects screen failed ^[1]^ |  |  |  |  |  |  |  | 129 (72.1) |
| Do not meet all eligibility  criteria |  |  |  |  |  |  |  | 75 (41.9) |
| Lost to follow-up |  |  |  |  |  |  |  | 5 (2.8) |
| Other |  |  |  |  |  |  |  | 39 (21.8) |
| Withdrawal by subject |  |  |  |  |  |  |  | 10 (5.6) |
| No. of subjects randomized and dosed | 10 (100.0) | 8 (100.0) | 8 (100.0) | 8 (100.0) | 8 (100.0) | 8 (100.0) | 40 (100.0) | 50 (100.0) |
| Completion of follow-up | 10 (100.0) | 8 (100.0) | 8 (100.0) | 7 (87.5) | 8 (100.0) | 8 (100.0) | 39 (97.5) | 49 (98.0) |
| Planned D113 follow-up | 4 (40.0) | 4 (50.0) | 1 (12.5) | 0 | 0 | 0 | 5 (12.5) | 9 (18.0) |
| Planned D253 follow-up | 6 (60.0) | 4 (50.0) | 7 (87.5) | 7 (87.5) | 8 (100.0) | 8 (100.0) | 34 (85.0) | 40 (80.0) |
| Discontinuation of follow-up | 0 | 0 | 0 | 1 (12.5) | 0 | 0 | 1 (2.5) | 1 (2.0) |
| Withdrawal by subject | 0 | 0 | 0 | 1 (12.5) | 0 | 0 | 1 (2.5) | 1 (2.0) |

Data presented are n (%). Percentages of [1] were calculated with “No. of subjects screened” as denominator. Percentages of others were calculated with “No. of subjects randomized and dosed” as denominator.

**Table S2. Dose Proportionality – Power Model of Pharmacokinetics Parameters**

|  | n | Estimate of Slope (90% CI) |
| --- | --- | --- |
| AUC_0-inf_, day·μg/mL | 27^a^ | 1.092 (0.9563, 1.2273) |
| AUC_last_, day·μg/mL | 40 | 1.182 (1.0855, 1.2787) |
| C_max_, μg/mL | 40 | 1.129 (1.0425, 1.2157) |

Dose range was 50-600 mg.

CI, confidence interval.

^a^ n = 27; 3, 7, 5, 4, and 8 subjects in the 50, 100, 200, 400, and 600 mg cohorts were included in the statistical analysis, respectively.

**Table S3. Summary of Anti-Drug Antibody (ADA)**

|  | Placebo  (n = 10) | SHR-1905 | | | | | | Overall  (n = 50) |
| --- | --- | --- | --- | --- | --- | --- | --- | --- |
|  |  | 50 mg  (n = 8) | 100 mg  (n = 8) | 200 mg  (n = 8) | 400 mg  (n = 8) | 600 mg  (n = 8) | Total  (n = 40) |  |
| Any ADA-positive sample | 2 (20.0) | 1 (12.5) | 0 | 1 (12.5) | 2 (25.0) | 3 (37.5) | 7 (17.5) | 9 (18.0) |
| Pre-existing ADA-positive subject | 1 (10.0) | 1 (12.5) | 0 | 0 | 0 | 0 | 1 (2.5) | 2 (4.0) |
| ADA-negative subject | 9 (90.0) | 8 (100.0) | 8 (100.0) | 7 (87.5) | 6 (75.0) | 5 (62.5) | 34 (85.0) | 43 (86.0) |
| ADA-positive subject | 1 (10.0) | 0 | 0 | 1 (12.5) | 2 (25.0) | 3 (37.5) | 6 (15.0) | 7 (14.0) |
| Treatment-induced ADA  positive subject | 1 (10.0) | 0 | 0 | 1 (12.5) | 2 (25.0) | 3 (37.5) | 6 (15.0) | 7 (14.0) |
| Treatment-boosted ADA  positive subject | 0 | 0 | 0 | 0 | 0 | 0 | 0 | 0 |

Subject with any ADA-positive was defined as subject with at least one baseline or post-baseline ADA-positive sample.

Pre-existing ADA-positive subject was defined as subject who had a baseline ADA-positive sample.

ADA-negative subject was defined as subject who was neither treatment-induced ADA-positive nor treatment-boosted ADA-positive.

ADA-positive subject was defined as subject who was treatment-induced ADA-positive or treatment-boosted ADA-positive.

Treatment-induced ADA-positive subject was defined as subject who had baseline ADA-negative sample and at least one post-baseline ADA-positive sample.

Treatment-boosted ADA-positive subject was defined as subject who had both baseline and post-baseline ADA-positive samples, and the titer of the post-baseline sample was equal to or more than 4-fold of the baseline titer.

ADA, anti-drug antibody.
