# Supplementary figures and images for: A phase 1, randomized, double-blind, placebo-controlled, dose escalation study to evaluate the safety, tolerability, pharmacokinetics and immunogenicity of SHR-1905, a long-acting anti-thymic stromal lymphopoietin antibody, in healthy subjects
Source: Front Pharmacol. 2024 Jul 15;15:1400696. doi: 10.3389/fphar.2024.1400696 (PMC11284144; doi:10.3389/fphar.2024.1400696)

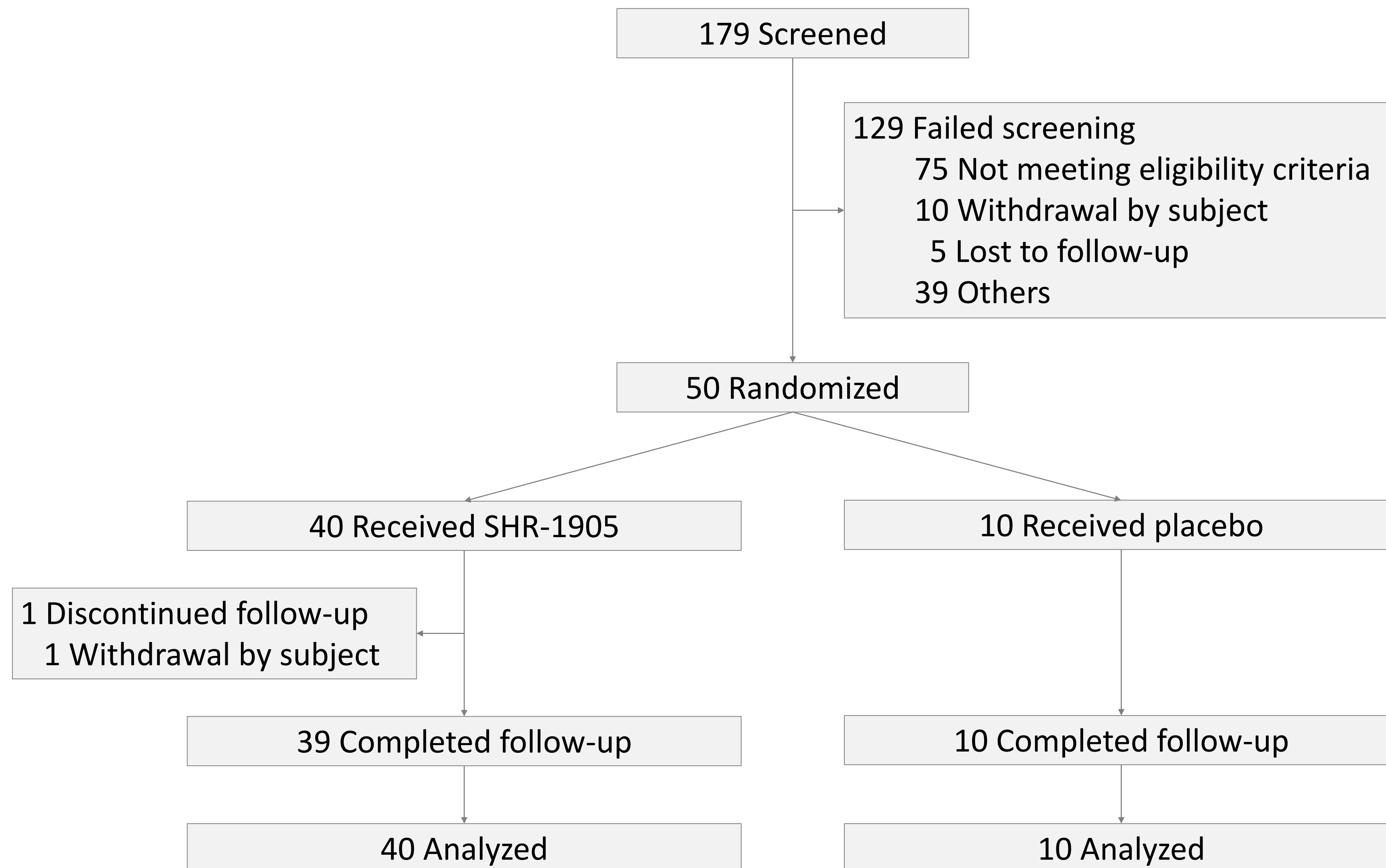

Supplement: Supplementary file 2 [file Image1.pdf]
